# Charge-transfer interface of insulating metal-organic frameworks with metallic conduction

**DOI:** 10.1038/s41467-022-35429-5

**Published:** 2022-12-12

**Authors:** Pooja Sindhu, K. S. Ananthram, Anil Jain, Kartick Tarafder, Nirmalya Ballav

**Affiliations:** 1 Department of Chemistry, Indian Institute of Science Education and Research, Dr. Homi Bhabha Road, Pune, 411 008 India; 2 Department of Physics, National Institute of Technology Karnataka, Surathkal, Mangalore, 575 025 India; 3 Solid State Physics Division, Bhabha Atomic Research Centre, Mumbai, 400085 India; 4 Homi Bhabha National Institute, Anushakti Nagar, Mumbai, 400094 India

**Keywords:** Chemistry, Metal-organic frameworks

## Abstract

Downsizing materials into hetero-structured thin film configurations is an important avenue to capture various interfacial phenomena. Metallic conduction at the interfaces of insulating transition metal oxides and organic molecules are notable examples, though, it remained elusive in the domain of coordination polymers including metal-organic frameworks (MOFs). MOFs are comprised of metal centers connected to organic linkers with an extended coordination geometry and potential void space. Poor orbitals overlap often makes these crystalline solids electrical insulators. Herein, we have fabricated hetero-structured thin film of a Mott and a band insulating MOFs via layer-by-layer method. Electrical transport measurements across the thin film evidenced an interfacial metallic conduction. The origin of such an unusual observation was understood by the first-principles density functional theory calculations; specifically, Bader charge analysis revealed significant accumulation and percolation of charge across the interface. We anticipate similar interfacial effects in other rationally designed hetero-structured thin films of MOFs.

## Abstract (web summary)

Electrical transport across hetero-structured thin films of band and Mott insulators is intriguing. Here, the authors report an emergence of interfacial metallic conduction across thin film of a Cu(I)- and Cu(II)-based metal-organic frameworks.

## Introduction

Interfaces of electrically insulating transition metal oxides (TMOs) are important experimental avenues for the realization of interesting interfacial phenomena like emergent magnetism, metallic conduction, high-mobility electron gas, culminating to superconductivity^1–7^. This is because of very rich electronic properties of the interfaces leading to various symmetry-breaking events of the order parameters; of particular mention is the broken inversion-symmetry at the interfaces of TMOs by the structure itself^8^. Typically, in TMOs, positively charged transition metal ion is coordinated to six negatively charged oxygen ions and thereby creating an octahedral crystal field around the metal ion, which lifts the five-fold degeneracy of the *d*-orbitals. Polar nature of the metal-oxygen bonds in combination with non-zero amplitude of the hopping matrix across an interface makes the correlated-electron behavior exotic when compared to the same in the bulk-phase—often leads to polar-catastrophe at the interfaces^9–11^. As for the organic molecules, metallic conduction at the interface was also realized though individual molecular counterparts in the respective bulk phases were found to be electrical insulators with band gap values of few electron volts^12^. Extended π-conjugation in the organic moieties was understood to be an important criterion for such molecular interfacial effects whereby one molecule can act as an electron donor and the other molecule can act as an electron acceptor—so called electron donor-acceptor system^12^. Structurally, TMO resembles metal-organic framework (MOF) in the sense that smaller oxygen ions in the former system are replaced by larger organic molecular ions (ligands) in the latter system, which brings permanent porosity in the crystalline solid framework^13^. Also, MOFs are often found to be electrical insulators likewise TMOs^14^.

In the last decade, design strategies have been implemented to confer reasonable electrical conductivity in MOFs, and the electrical transport has been understood as follows: (i) through-bond, (ii) through-space, (iii) extended conjugation, (iv) redox-hopping, and (v) guest-promoted transport^15,16^. In general, the charge transport pathways in electrically conducting MOFs can be described by either band transport or hopping transport mechanism—nicely summarized by Dincă et al.^15,16^: in the former, formation of continuous energy bands due to strong interaction between sites enables delocalization of charge carriers while in the latter, charge carriers are localized on discrete or non-bonded sites and hop between the neighboring sites in MOFs. As for the guest promoted transport, an exemplary work by Allendorf et al.^17^ in the domain of thin films of MOFs is the doping of redox-active molecule TCNQ (7,7,8,8-tetracyanoquinodimethane) molecule into the framework of Cu(II)-BTC (BTC = 1,3,5-benzenetricarboxylate) which resulted in the remarkable enhancement of electrical conductivity value by six orders of magnitude (~10^−8^ S/cm to ~10^−2^ S/cm). The conductivity enhancement has been assigned to be due to an efficient orbital-overlap (*d*_x_^2^_−__y_^2^ → π* and π → *d*_x_^2^_−y_^2^) leading to lowering of the band gap value from 3.61 eV for Cu-BTC to 1.76 eV for TCNQ@Cu-BTC^17,18^. Mechanistic understanding of such an interesting observation was initially found to be the generation of conducting paths in Cu-BTC framework due to bridging of two Cu paddle wheel dimers via two geminal nitrile groups of TCNQ molecule, facilitating the charge-transfer involving highest occupied molecular orbital (HOMO) of Cu-BTC (localized on the copper atom) and lowest unoccupied molecular orbital (LUMO) of TCNQ (localized on the π-orbital)^17–22^. An extended hopping model (or molecular superexchange) for charge transport in the TCNQ@Cu-BTC system has also been proposed to explain such an unusual enhancement in the electrical conductivity^22^.

**Fig. 1:**
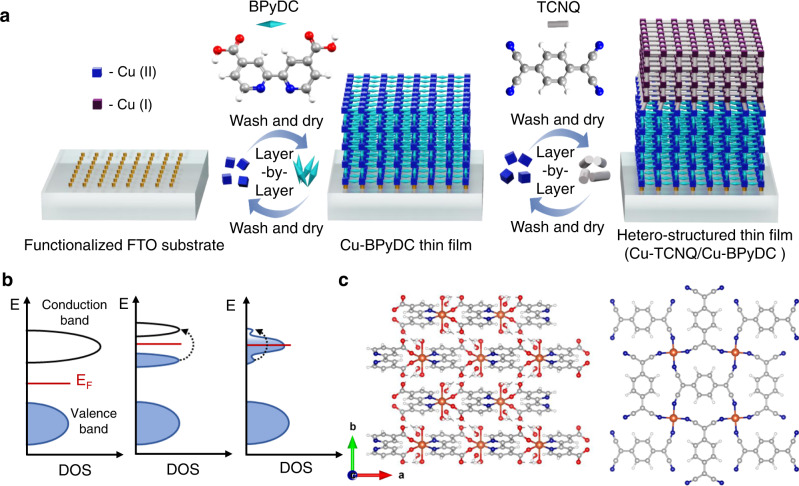
Schematics of the growth of hetero-structured thin film of electrically insulating MOFs. **a** Layer-by-layer (LbL) growth of Cu-TCNQ thin film on top of Cu-BPyDC thin film via IRR leading to hetero-structured Cu(I)-TCNQ/Cu(II)-BPyDC thin film. **b** Band diagram schemes of a band insulator (left), a Mott insulator (middle), and their interface (right). Fermi level (E_F_) is marked by red line. **c** Energy optimized structures of Cu-BPyDC (left) and Cu-TCNQ (right) (view from *ab* plane) where carbon, hydrogen, nitrogen, oxygen, and copper atoms are represented by colors gray, white, blue, red, and orange, respectively.

In view of poor electrical conductivity of MOFs coupled with fabrication challenges of downsizing these materials into crystalline thin film configurations, investigations on electron transport across hetero-structured thin films of MOFs are rarely explored^23^, except the recent studies on the realization of p-n hetero-junction diodes^24,25^. Herein, we present an emergence of metallic conduction at the interface of a Mott and a band insulating MOFs. Highly-crystalline hetero-structured thin films of MOFs on fluorine-doped tin oxide (FTO) substrate were fabricated by employing simple layer-by-layer (LbL) method. Specifically, we have grown thin film of Cu(II)-BPyDC (BPyDC = 2,2′-bipyridene-4,4′-dicarboxylate), which is assigned as a band insulator and on top of it, we have deposited thin film of Cu(I)-TCNQ (TCNQ = 7,7,8,8-tetracyanoquinodimethane) which is a Mott (electron-electron interaction) insulator^26^ resulting in the generation of Cu(I)-TCNQ/Cu(II)-BPyDC hetero-structured thin film (Fig. 1). Fabrication of such Cu(II)-Cu(I) (3*d*^9^–3*d*^10^) interface is attributed to the interfacial reduction reaction (IRR)^24,27,28^ whereby solution of Cu(II)(OAc)_2_ was consistently introduced as the metal ion precursor during two consecutive LbL growth with the BPyDC and TCNQ ligands.

## Results

**Fig. 2:**
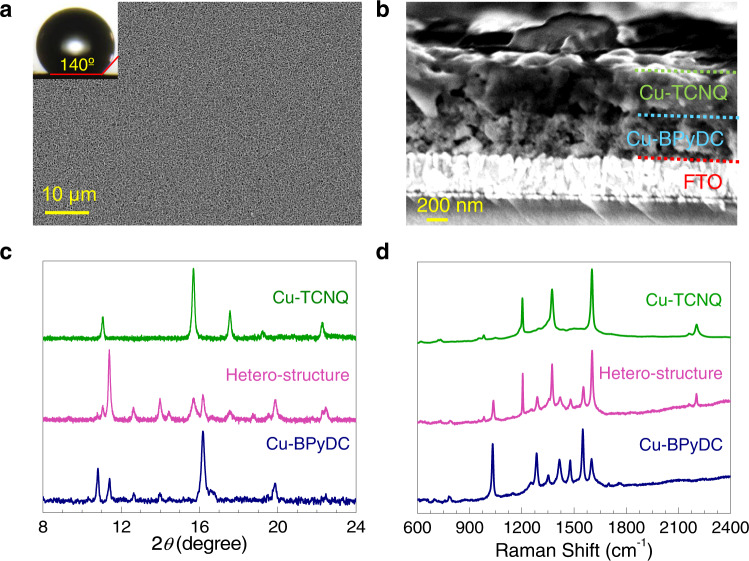
Morphology and structural analysis of pristine and hetero-structured thin films. **a** FESEM image (top-view) showing highly-uniform coverage of the hetero-structured thin film (inset: an optical image of the contact angle of water). **b** Cross-sectional FESEM image of hetero-structured Cu-TCNQ/Cu-BPyDC thin film with identifying distinctive layers of FTO, Cu-BPyDC, and Cu-TCNQ (marked by dotted lines). **c** Out-of-plane XRD patterns of pristine Cu-BPyDC (blue), pristine Cu-TCNQ (green) and hetero-structured Cu-TCNQ/Cu-BPyDC (pink) thin films. **d** Raman spectra on pristine Cu-BPyDC (blue), pristine Cu-TCNQ (green), and hetero-structured Cu-TCNQ/Cu-BPyDC (pink) thin films.

Field-emission scanning electron microscopy (FESEM) images revealed formation of highly-uniform pristine Cu-TCNQ, pristine Cu-BPyDC, and hetero-structured Cu-TCNQ/Cu-BPyDC thin films (Fig. 2a and Supplementary Fig. 1). Distinctive morphological patterns of the micro-crystallites of Cu-TCNQ and Cu-BPyDC were visible in the respective zoomed-in FESEM images (Supplementary Fig. 1). In cross-sectional FESEM images of the Cu-TCNQ/Cu-BPyDC system, layers of FTO, Cu-BPyDC, and Cu-TCNQ could be clearly identified thereby attesting the successful formation of hetero-structured thin film of an approximate thickness of 1 μm (Fig. 2b). Also, top-view zoomed-in FESEM image on the Cu-TCNQ/Cu-BPyDC thin film revealed Cu-TCNQ crystallites with characteristic morphology as expected^24,29^ (Supplementary Fig. 1). Water contact angle values of 140 ± 5° on both pristine Cu-TCNQ and pristine Cu-BPyDC thin films suggested highly-hydrophobic surfaces so as the surface of the hetero-structured Cu-TCNQ/Cu-BPyDC thin film (Fig. 2a and Supplementary Fig. 1)^29^.

Room-temperature out-of-plane X-ray diffraction (XRD) patterns of both pristine and hetero-structured thin films were recorded, which revealed the presence of characteristics diffraction peaks of Cu-TCNQ^30,31^ and Cu-BPyDC^32,33^ in the Cu-TCNQ/Cu-BPyDC system, and, in general, endorsed formation of highly-crystalline thin films via LbL (Fig. 2c). XRD pattern of the Cu-TCNQ thin film matched well with the simulated XRD pattern and confirmed the presence of a single-phase system (Supplementary Figs. 2 and 3). As for the Cu-BPyDC thin film, the preferred orientation was determined by looking at the peak intensities and was applied during the Rietveld refinement which confirmed the presence of mixed-phase system with one major (assigned as phase-I) and one minor (assigned as phase-II) components (Supplementary Figs. 2, 4 and 5). Rietveld refinement of the XRD pattern of the hetero-structured Cu-TCNQ/Cu-BPyDC thin film was performed considering three-phase system (two-phase of Cu-BPyDC and one-phase of Cu-TCNQ) and only scale factor was varied whereby a reasonably good match between the experimental and calculated patterns was achieved (Supplementary Fig. 2). Raman spectra of pristine Cu-TCNQ, pristine Cu-BPyDC and hetero-structured Cu-TCNQ/Cu-BPyDC thin films were compared and characteristic vibrational signatures of Cu-TCNQ and Cu-BPyDC were found to be clearly present in the Cu-TCNQ/Cu-BPyDC system^24,29,34^ (Fig. 2d). Specifically, the peak at ~1374 cm^−1^ corresponding to the C–CN stretching mode represented Cu-TCNQ^29^ while the peak at ~1033 cm^−1^ corresponding to the skeletal mode of the bipyridine moiety represented Cu-BPyDC^34^ (Fig. 2d). Therefore, Raman spectra along with FESEM and XRD data confirmed the successful fabrication of hetero-structured Cu-TCNQ/Cu-BPyDC thin film with retention of structural integrity. Oxidation states of Cu ions as (II) and (I) in Cu-BPyDC and Cu-TCNQ, respectively, were confirmed by X-ray photoelectron spectroscopy (XPS); upon growing few layers of Cu-TCNQ (5 cycles) on top of the pristine Cu-BPyDC thin film, clear cut signatures of Cu(I) and Cu(II) were identified in the Cu 2*p* XPS signal thereby justifying the formation of 3*d*^10^-3*d*^9^ Cu(I)/Cu(II) interface in the hetero-structured Cu-TCNQ/Cu-BPyDC system (Supplementary Fig. 6). Specifically, Cu 2*p*_3/2_ photoemission signals at binding energy values of ~932.5 and ~934.2 eV with concomitant absence and presence of satellite spectral features, were characteristics of Cu-TCNQ and Cu-BPyDC, respectively^27,28^ (Supplementary Fig. 6).

**Fig. 3:**
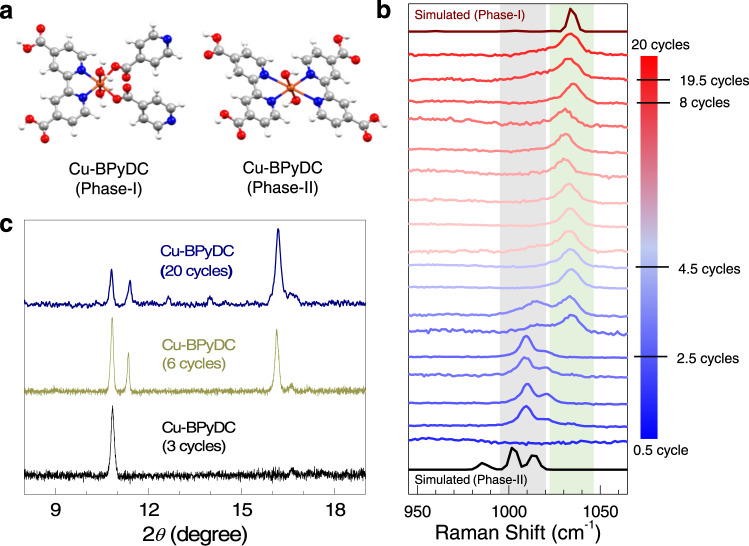
Identifying phase-I and phase-II structures of Cu-BPyDC. **a** Characteristic molecular fragments isolated from phase-I (left panel) and phase-II (right panel) structures of Cu-BPyDC and subsequently used for the simulation of Raman spectral signatures. **b** Experimental Raman spectra recorded during the growth of Cu-BPyDC thin film, at each step of LbL and up to 20 cycles; and compared with the theoretically simulated Raman spectra for phase-I and phase-II structures of Cu-BPyDC. **c** Out-of-plane XRD patterns recorded during the growth (3 cycles, 6 cycles, and 20 cycles) of pristine Cu-BPyDC thin film.

To understand the origin of mixed-phase in the Cu-BPyDC system, we have recorded cycling-dependent Raman spectra, compared with the simulated Raman spectra obtained from density functional theory (DFT) calculations, and recorded cycling dependent out-of-plane XRD patterns (Fig. 3). In the simulation of Raman spectra, we have considered characteristic molecular fragments of phase-I and phase-II structures of the Cu-BPyDC system (Fig. 3a). Specifically, in the phase-I structure, along with two water molecules, two oxygen and two nitrogen from three different BPyDC ligands were found to be coordinated to Cu(II) ion (Supplementary Fig. 4) and in the phase-II structure, along with two hydroxyl groups, four nitrogen from two different BPyDC ligand were coordinated to Cu(II) ion (Supplementary Fig. 5). Due to distinctive coordination environments around Cu(II), vibrational modes associated with the BPyDC ligand are expected to be significantly different. Experimentally, in the Raman spectra (Fig. 3b), during the initial growth cycles (up to 2.5 cycles) of Cu-BPyDC, one major peak at 1009 cm^−1^ was observed along with one minor peak at 1020 cm^−1^. After 2.5 cycles, a new peak was found to emerge at 1033 cm^−1^ along with the small co-existence of the aforementioned peaks which could be considered as the transition point for the onset of a new phase. Beyond 3.5 cycles, a single peak at 1033 cm^−1^ was consistently observed up to 20 cycles. With the help of the simulated Raman spectra, the peak at 1033 and 1009 cm^−1^ are assigned to be characteristic of phase-I and phase-II structures of the Cu-BPyDC system, respectively. Raman spectra also endorsed the phase-I structure as the major component (16 cycles of growth) and phase-II structure as the minor component (4 cycles of growth) within the Cu-BPyDC thin film thereby supporting the Rietveld refinement of the XRD patterns. To further strengthen our claim on mixed-phase of Cu-BPyDC, we have recorded cycling dependent out-of-plane XRD patterns during the growth of the pristine Cu-BPyDC thin film; after 3 cycles of growth, presence of phase-II structure was exclusively detected and only after 6 cycles (as well as 20 cycles) appearance of phase-I along with phase-II was clearly identified (Fig. 3c). Cycling-dependent XPS data complemented the Raman data by showing distinctive Cu 2*p* photoemission signals during the growth of Cu-BPyDC thin film, confirming the presence of almost exclusive presence of Cu(II) in the system (Supplementary Fig. 7).

**Fig. 4:**
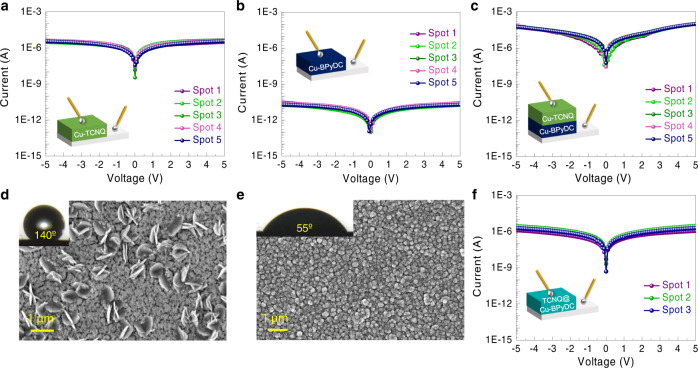
Electrical transport measurements on pristine and hetero-structured thin films. **a** Cross-plane *I*–*V* characteristics recorded on five different spots (marked as 1 to 5) in pristine Cu-TCNQ thin film (inset: schematic of measurement with top and bottom eutectic GaIn contacts). **b** Cross-plane *I*–*V* characteristics recorded on five different spots (marked as 1 to 5) in pristine Cu-BPyDC thin film (inset: schematic of measurement with top and bottom eutectic GaIn contacts). **c** Cross-plane *I*–*V* characteristics recorded on five different spots (marked as 1 to 5) in the Cu-TCNQ/Cu-BPyDC thin film (inset: schematic of measurement with top and bottom eutectic GaIn contacts). **d** FESEM image (top-view) showing the initial growth (few LbL cycles) of Cu-TCNQ crystallites with distinctive nanodisc-like morphology on top of the Cu-BPyDC thin film (inset: an optical image of the contact angle of water). **e** FESEM image (top-view) showing morphological features of TCNQ@Cu-BPyDC thin film (inset: an optical image of the contact angle of water). **f** Cross-plane *I*–*V* characteristics recorded on three different spots (marked as 1 to 3) in the TCNQ@Cu-BPyDC thin film (inset: schematic of measurement with top and bottom eutectic GaIn contacts).

Electrical transport properties of pristine Cu-TCNQ, pristine Cu-BPyDC and hetero-structured Cu-TCNQ/Cu-BPyDC thin films were evaluated by recording current–voltage (*I*–*V*) profiles (in-plane and cross-plane modes) at room-temperature (300 K). *I*–*V* profiles for pristine Cu-TCNQ thin film were found to be similar in both in-plane and cross-plane modes with conductance value of ~6 × 10^−7^ S (Fig. 4a and Supplementary Fig. 8) and so as the *I*–*V* profiles for pristine Cu-BPyDC thin film with conductance value of ~4 × 10^−12^ S (Fig. 4b and Supplementary Fig. 8). As for the hetero-structured Cu-TCNQ/Cu-BPyDC thin film, conductance value was found to be ~5 × 10^−7^ S in the in-plane mode—almost identical to the value observed for the pristine Cu-TCNQ thin film, as expected (Supplementary Fig. 8). Interestingly however, in the cross-plane mode, a conductance value of ~1 × 10^−5^ S for the hetero-structured Cu-TCNQ/Cu-BPyDC thin film was realized which is even higher and markedly-higher than the values of pristine Cu-TCNQ and Cu-BPyDC thin films, respectively (Fig. 4c). Notably, *I*–*V* profiles of pristine Cu-TCNQ, pristine Cu-BPyDC and hetero-structured Cu-TCNQ/Cu-BPyDC thin films found on various spots in the same sample (Fig. 4) and across different batches of samples in both in-plane and cross-plane modes were observed to be consistent (Supplementary Figs. 9 and 10). Also, our solution processed interface was realized to be ambient-stable over months—retaining almost identical *I*–*V* profiles and reflecting a robust electrical transport property (Supplementary Fig. 11).

One possibility of such a prominent enhancement in electrical conductance value could be due to the fact that TCNQ diffused inside Cu-BPyDC thin film during LbL growth of hetero-structured Cu-TCNQ/Cu-BPyDC thin film, as was elegantly demonstrated earlier by Allendorf et al.^17^ for the tuning of electrical conductivity upon molecular doping of Cu-BTC by TCNQ. Such an impregnation of TCNQ resulted in successful transformation of an insulating MOF (Cu-BTC) to a semiconductor material (TCNQ@Cu-BTC)^17,18,21,22^. Motivated by these remarkable observations, we have deliberately impregnated Cu-BPyDC thin film with TCNQ following our standardized procedure^35^ (similar to the one reported by Allendorf et al.^17^) and performed a comparative assessment of TCNQ@Cu-BPyDC thin film with hetero-structured Cu-TCNQ/Cu-BPyDC thin film. It is worth to mention here that in a recent study^24^, we were able to successfully fabricate hetero-structured Cu-TCNQ/Cu-BTC thin film by carefully avoiding the impregnation of TCNQ in Cu-BTC framework, and the same approach has been adopted in the present work i.e., upon reducing the reaction time of the initial few LbL growth cycles of Cu-TCNQ on top of Cu-BPyDC thin film. As revealed by FE-SEM images, after one LbL growth cycle, Cu-TCNQ crystallites with characteristic morphology were clearly visible on top of Cu-BPyDC thin film (Fig. 4d) which is very different from the morphological features on TCNQ@Cu-BPyDC thin film (Fig. 4e). The surface wettability after one LbL growth cycle of Cu-TCNQ on Cu-BPyDC thin film was markedly different from that of TCNQ@Cu-BPyDC thin film, as indicated by the water contact angle values of ~140 ± 5° (Fig. 4d) and ~55 ± 5° (Fig. 4e), respectively.

The in-plane *I*–*V* profiles for TCNQ@Cu-BPyDC thin film (Supplementary Figs. 8 and 9) with conductance value of ~6 × 10^−7^ S were found to be similar to those of hetero-structured Cu-TCNQ/Cu-BPyDC (and pristine Cu-TCNQ) thin film. The cross-plane *I*–*V* profiles for TCNQ@Cu-BPyDC thin film with conductance value of ~3 × 10^−7^ S (Fig. 4f and Supplementary Fig. 10) were noticeably different from those of hetero-structured Cu-TCNQ/Cu-BPyDC thin film. We have also estimated the resistivity values from the respective current–density (*J*)–*V* plots (Supplementary Fig. 12). The resistivity values of TCNQ@Cu-BPyDC thin film (~1 × 10^6^ Ωm) and earlier reported TCNQ@Cu-BTC thin film (~5 × 10^7^ Ωm)^22^ were found to be more than hundred-fold and thousand-fold higher, respectively, than the resistivity value of hetero-structured Cu-TCNQ/Cu-BPyDC thin film (~1 × 10^4^ Ωm). Thus, such a pronounced increment in the cross-plane electrical conductance of hetero-structured Cu-TCNQ/Cu-BPyDC thin film, specifically in comparison to pristine Cu-BPyDC thin film, could not be simply attributed to the pore impregnation (by TCNQ) effect and suggested an emergence of an interfacial phenomenon in the hetero-structured thin film viz. metallic conduction.

**Fig. 5:**
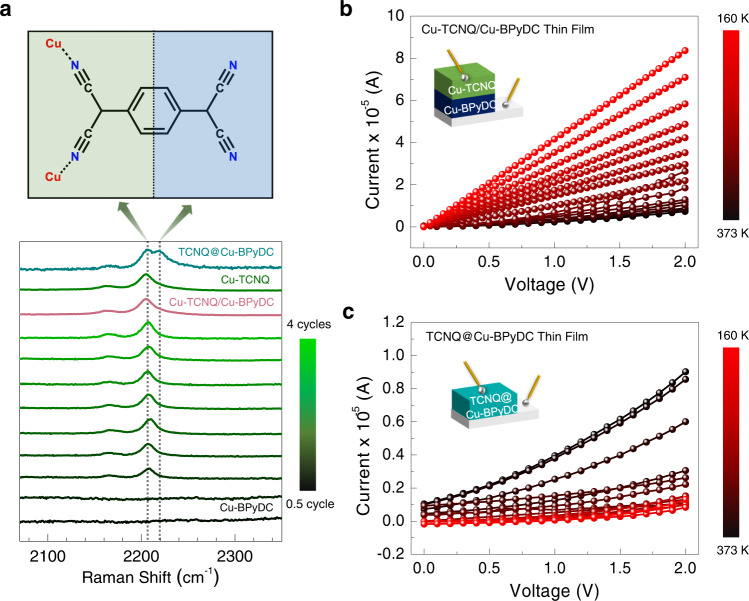
Eliminating the effect of molecular doping. **a** Raman spectra recorded during the growth of Cu-TCNQ on top of Cu-BPyDC thin film (at each step of LbL and up to 4 cycles). Raman spectra of pristine Cu-TCNQ and TCNQ @Cu-BPyDC thin films are also provided for a direct comparison. CN groups of TCNQ will have different vibrational signatures if coordinated to metal ion (like Cu in the present study)—as schematically shown and highlighted by blue and green colors. Positions of the vibrational bands are marked by dotted lines. **b** Current-voltage (*I*–*V*) profiles at different temperatures, ranging from 160 to 373 K, on the hetero-structured Cu-TCNQ/Cu-BPyDC thin film in the cross-plane mode. **c** Current–voltage (*I*–*V*) profiles at different temperatures (160–373 K) on the TCNQ@Cu-BPyDC thin film in the cross-plane mode.

Raman spectra of TCNQ@Cu-BPyDC and hetero-structured Cu-TCNQ/Cu-BPyDC thin films were distinctively different (Fig. 5a). Specifically, in addition to the vibrational band at ~2205 cm^−1^, appearance of an additional vibrational band at ~2220 cm^−1^ in the Raman spectrum of TCNQ@Cu-BPyDC thin film endorsed two non-equivalent bonding environments of TCNQ with two nitrile groups bonded to the Cu(II) ions while other two nitrile groups remained unbind, as were earlier shown by Allendorf et al.^17^ for the TCNQ@Cu-BTC thin film and by our group^35^ for the TCNQ@Cu-BTEC (BTEC = 1,2,4,5-benzenetetracarboxylate) thin film. In the Raman spectrum of our hetero-structured Cu-TCNQ/Cu-BPyDC thin film, only one vibrational band at ~2205 cm^−1^ appeared which is characteristic of Cu-TCNQ (Supplementary Fig. 13) and absence of the vibrational band at ~2220 cm^−1^ evidenced no diffusion or impregnation of TCNQ into Cu-BPyDC layers. Further, Raman spectra were recorded during the initial growth of Cu-TCNQ on top of Cu-BPyDC thin film, at each step and up to four consecutive cycles of LbL (Fig. 5a and Supplementary Fig. 13). Notably, vibrational band at ~2220 cm^−1^, serving as the fingerprint of a molecular doping phenomenon, was consistently absent (Fig. 5a). Therefore, our cycling-dependent Raman spectroscopic data clearly ruled out the possibility of any diffusion or impregnation of TCNQ into Cu-BPyDC layers, even at the microscopic level and our hetero-structured thin film is not a system like Cu-TCNQ/TCNQ@Cu-BPyDC. Finally, temperature dependence of the electrical transport property was evaluated by recording the *I*–*V* profiles, in the cross-plane mode, at various temperature, ranging from low-temperature (160 K) to high-temperature (373 K). In the hetero-structured Cu-TCNQ/Cu-BPyDC thin film, a gradual increase in the conductance/conductivity value upon decreasing the temperature from 373 K down to 160 K (Fig. 5b) confirmed metallic conduction. On the contrary, an opposite trend was realized with the TCNQ@Cu-BPyDC thin film where conductance/conductivity value gradually increased upon increasing the temperature from 160 to 373 K (Fig. 5c)—as expected for a semiconducting system like the well-celebrated TCNQ@Cu-BTC system^17^.

## Discussion

**Fig. 6:**
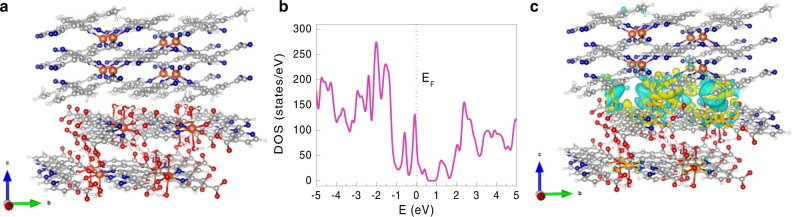
Probing electronic structure of the interface by DFT calculations. **a** Energy optimized structure of the interface involving Cu-TCNQ (top) and phase-I of Cu-BPyDC (bottom) where carbon, hydrogen, nitrogen, oxygen, and copper atoms are represented by colors gray, white, blue, red, and orange, respectively (view from *bc* plane). **b** Total density of state (DOS) for the interface structure (pink), a new large peak at the Fermi energy (*E*_F_, marked by dotted black line) appears due to significant charge redistribution and strong hybridization at the interface structure. **c** Percolated charge density due to the formation of the interface is presented where electron accumulation and electron depletion are represented by yellow and cyan isosurfaces, respectively (view from *bc* plane).

To understand the origin of such an interesting interfacial effect, numerical simulations based on density functional theory (DFT) with additional Hubbard interaction (+U) were performed on Cu-TCNQ, Cu-BPyDC, and hetero-structured Cu-TCNQ/Cu-BPyDC systems^36–38^. The bulk structures of Cu-TCNQ (C_48_H_16_N_16_Cu_4_) and phase-I Cu-BPyDC (C_48_H_56_N_8_O_32_Cu_4_) were modeled by using experimentally obtained crystal structure data^31,32^ and duly optimized (Fig. 1c and Supplementary Table 1). After performing bulk geometry optimization of Cu-TCNQ and Cu-BPyDC, an interface structure was constructed by modeling slabs and stacking them along (001) crystal direction of Cu-BPyDC (Fig. 6a). We found that the (001) crystal direction was most favorable for the formation of the Cu-TCNQ/Cu-BPyDC (C_236_H_200_N_48_O_64_Cu_16_) interface as the surface energies as well as the lattice mismatch between slabs were minimum. Also, strain on the individual slabs of Cu-TCNQ and Cu-BPyDC was found to be minimum when stacking them along (001) crystal direction of Cu-BPyDC. Our calculations show that Cu-TCNQ and Cu-BPyDC systems have band gap values of 2.1 and 2.7 eV respectively (Supplementary Fig. 14), corroborating the optical band gap values obtained from the respective solid-state UV–vis absorption spectra viz. Tauc plots^24^ (Supplementary Fig. 15). Remarkably, interface was found to be metallic with a finite density of states (DOS) at the Fermi level (*E*_F_) (Fig. 6b). To recognize this unusual behavior, the difference in charge density due to the formation of the interface was estimated by using the formula *ρ* = *ρ*(AB) − *ρ*(A) − *ρ*(B), where *ρ*(A), *ρ*(B) and *ρ*(AB) are the charge densities for Cu-TCNQ, Cu-BPyDC, and Cu-TCNQ/Cu-BPyDC interface, respectively. Significant charge accumulation at the interface is clearly visible in the charge–density plot (Fig. 6c and Supplementary Fig. 16). Specifically, Bader charge analysis^39^ was carried out using Henkelman’s group program^40–42^. Bader charges are charges of involved atoms defined by an infinitesimal volume of minimal gradient around each atom (Supplementary Table 2). Charge transfer in the system is estimated to be equal to [Bader charge of the interface (Cu(I)/Cu(II))—Bader charge of Cu(I) structure—Bader charge of Cu(II) structure].

To find out the charge accumulation at the interface, the distribution of the Bader charge on twenty-eight atoms at the interface is calculated. In this calculation we were only able to specify which atoms are affected and Bader charge analysis was unable to provide the orbital nature of the transferred charges. The details of charges transferred from different atoms at the interface are also summarized (Supplementary Table 3). The negative Bader charge difference indicates the charge depletion and the positive value indicates the charge accumulation at the atom center (indicated by cyan and yellow isosurfaces, respectively in Fig. 6c). According to our calculations, an approximate net charge of 0.0026 e/Å^3^ (or total of 3.52 e per interfacial unit cell) was accumulated at the interface and the charges were estimated to percolate about 7 Å away from the interface. Thus, DFT + U calculations combined with experimental observations evidenced an interfacial metallic conduction. Notably, our cycling-dependent XRD patterns (Fig. 3c) revealed that with increasing the number of cycles, phase-I structure of Cu-BPyDC became dominant over phase-II and, therefore, primarily contributing to the interfacial effects in the hetero-structured Cu-TCNQ/Cu-BPyDC thin film. We have additionally performed similar DFT (+U) calculations upon taking the same crystal structure of Cu-TCNQ (C_48_H_16_N_16_Cu_4_) but with phase-II crystal structure of Cu-BPyDC (C_96_H_64_N_16_O_44_Cu_10_) obtained from the Rietveld refinement (Supplementary Table 4). After performing geometry optimization of both the bulk structures, an interface structure was constructed by stacking them along (001) crystal direction of Cu-BPyDC (Supplementary Fig. 17). Bader charge analysis of the Cu-TCNQ/Cu-BPyDC (C_480_H_224_N_128_O_88_Cu_32_) interface was carried out (Supplementary Tables 5 and 6) likewise the aforementioned system, and in comparison to phase-II structure of Cu-BPyDC, better charge accumulation and percolation across the interface was realized with the phase-I structure of Cu-BPyDC. Remarkably, metallic interface was consistently observed (Supplementary Fig. 17), thereby suggesting that the interfacial charge-transfer chemistry of insulating Cu(II)-BPyDC and Cu(I)-TCNQ MOFs is indeed engrossing and can have far-reaching implications.

Charge-transfer phenomenon has been observed earlier in MOFs upon introducing various design strategies such as incorporation of redox-active guest molecules, integration of mixed donor-acceptor linkers in a single framework, post-synthetic modifications, and many more. An elegant example has already been discussed where redox-active TCNQ molecules were loaded inside the pores of Cu-BTC and a dramatic enhancement in the electrical conductivity was observed, which originated from host-guest charge-transfer interaction^17–22^. The photo-physical effects on infiltration of α,ω-dihexylsexithiophene (DH6T) and [6,6]-phenyl-C_61_-butyric acid methyl ester (PCBM) inside the pores of MOF-177 (ZnO_4_(BTB)_2_; BTB = 1,3,5-benzenetribenzoate) has been studied with different sample configurations, and consistently, an efficient quenching of MOF-177 luminescence was observed via host-guest Förster resonance energy transfer (FRET) mechanism^43^. Also, host–guest charge-transfer was assigned to occur due to the unique special arrangements allowing overlapping of p-orbitals of the BTB linkers of MOF host with the fullerene ring of PCBM guest^43^. MOF with mixed donor–acceptor linkers of donor TTFTB (tetrathiafulvalene tetrabenzoate) and acceptor bpmNDI [N,N’-bis(4-pyridylmethyl)-1,4,5,8-naphthalene diimide] exhibited high-degree of charge-transfer from TTF to NDI resulting in a broad absorption in the near infra-red (NIR) region^44^. TTF and NDI moieties were found to interact in a face-to-face manner in the framework with dihedral angle of ca. 8.17° and ca. 3.45 Å distance apart facilitating donor-acceptor charge-transfer and π–π interactions^44^. Wavelength-dependent energy and charge-transfer in a MOF, post-synthetically modified by photo-active porphyrin moiety, also enabled realization of an artificial light-harvesting system^45^.

Core-shell hetero-structures of Prussian blue-based MOF (core) and porphyrin-based MOF (shell) were developed for the enhancement of photocatalytic properties whereby photo-inspired electrons transfer process can be facilitated while suppressing the recombination of electrons and holes^46^. Hetero-structured core-shell system of two Ti-based MOFs also helped to realize multi-stepwise charge transfer during photo-catalytic hydrogen evolution^47^. One-dimensional micro-rods of Eu-BTC and Tb-BTC were successfully grown in tri-block hetero-structured configurations for the development of photonic barcodes^48^. Additionally, A-B-A type tri-layer hetero-structured thin film was fabricated by LbL technique and Dexter transfer of triplet excitons over the heterojunction demonstrated electron transfer across the layers^49^. AB-type hetero-structured thin films of lanthanides (Eu and Tb)-based MOFs with different compositional ratios were fabricated just by varying the growth cycles in LbL and tunable emission was achieved^50^. Also, ABC-type tri-layer hetero-structured thin films of Eu, Gd, and Tb-based MOFs exhibited white-light emission^51^. Interestingly, however, none of the reports dealing with the charge-transfer phenomenon in MOFs led to observation of metallic conduction in the system, in particular for the hetero-structured thin films of MOFs. Only two reports on the electrical transport across hetero-structured MOF thin films have been reported till date. In the first report, AB- and BA-type hetero-structured thin films of Cu-BTC and Cu-TCNQ were successfully fabricated by LbL technique and rectification of electrical current in the cross-plane mode was consistently observed with similar on-off ratio, which was assigned to be due to generation of p-n and n-p junction diode interfaces^24^. In the second report, AB- and BA-type hetero-structured thin films of p-type Cu_2_(adc)_2_(dabco) and n-type C_60_@Cu_2_(bdc)_2_(dabco) (adc = 9,10-anthracene dicarboxylate, bdc = 1,4-benzene dicarboxylate and dabco = 1,4-diazabicyclo[2.2.2]octane) MOFs were fabricated by LbL technique and distinctive on-off ratios for the p-n and n-p interfaces were realized^25^. Therefore, in conjunction with charge/energy transfer across MOF hetero-structures, our findings on metallic conduction across the interface of insulating MOFs will certainly open up possibilities in the field.

In summary, we have presented an interface of Cu(I)(3*d*^10^) and Cu(II)(3*d*^9^) ions-based insulating MOFs coordinated to TCNQ and BPyDC ligands, respectively. Electrical conductance value across the hetero-structured Cu-TCNQ/Cu-BPyDC thin film was found to be more than ten-fold and million-fold higher than the values of pristine Cu-TCNQ and Cu-BPyDC thin films, respectively. Raman spectral signatures ruled out the possibility of any pore impregnation of Cu-BPyDC layers by TCNQ in the hetero-structured Cu-TCNQ/Cu-BPyDC thin film. Such a pronounced enhancement in the electrical conductivity was attributed to be due to an interfacial metallic conduction and was duly endorsed by the temperature dependence of the *I*–*V* profiles. Electrical transport measurements were further substantiated by numerical simulations based on DFT (+U) calculations revealing a strong interlayer hybridization and charge-transfer leading to an overlap of conduction band (CB) and valence band (VB) in the Cu-TCNQ/Cu-BPyDC interface (even consistent with different crystal structures of Cu-BPyDC) while finite gaps between CB and VB for both Cu-TCNQ and Cu-BPyDC. Our results clearly open an avenue for the development of hetero-structured MOF-based thin film electronic devices in particular, because most often MOFs are electrical insulators.

## Methods

### Chemicals

Copper acetate [Cu(OAc)_2_.H_2_O], 7,7,8,8-tetracyanoquinodimethane (TCNQ), 2,2′-bipyridene-4,4′-dicarboxylic acid (H_2_BPyDC), Fluorine doped tin oxide (FTO) coated glass substrate (surface resistivity ~ 7 Ω/sq), and dimethylformamide (DMF) were purchased from Sigma-Aldrich and ethanol from Merck and used as such, without any further purification except FTO coated glass substrate.

### Functionalization of FTO substrate

FTO substrate was first cleaned by sonication in acetone, ethanol and water separately for 15 min each, washed with the Milli-Q water and dried under the N_2_ flow. Thereafter, the FTO substrate was functionalized by treating it with a mixture of conc. KOH (2 mmol) aqueous solution and H_2_O_2_ (30%) with the volume ratio of 3:1 at 80 °C for 30 min and then rinsed with the Milli-Q water and dried under the stream of N_2_ gas^52^.

### Fabrication of pristine Cu-BPyDC thin film

Functionalized FTO substrate was immersed in 1 mM Cu(OAc)_2_.H_2_O solution in DMF kept at 330 K for 30 min and then washed with DMF solvent to remove the uncoordinated metal ions followed by drying step with N_2_ gas and subsequently in 1 mM BPyDC solution in DMF kept at 330 K for 30 min (washed with DMF in between followed by drying with stream of N_2_ gas) to complete one LbL cycle. 20 cycles of LbL were performed to get a uniform Cu-BPyDC thin film (thickness values were estimated to be approximately 400 nm)^24^.

### Fabrication of pristine Cu-TCNQ thin film

FTO substrate was immersed in 1 mM ethanolic solution of Cu(OAc)_2_.H_2_O kept at 330 K for 30 min followed by washing with ethanol and subsequently in 1 mM ethanolic solution of TCNQ kept at 330 K for 30 min (washed with ethanol in between followed by drying with stream of N_2_ gas) to complete one LbL cycle. 20 cycles of LbL were performed to get a uniform Cu-TCNQ thin film (thickness values were estimated to be approximately 600 nm)^24^.

### Fabrication of Cu-TCNQ/Cu-BPyDC hetero-structured thin film

Prefabricated pristine Cu-BPyDC thin film was subsequently dipped into ethanolic solutions of Cu(OAc)_2_.H_2_O and TCNQ (five minutes in each solution at an elevated temperature and washed with ethanol in between followed by drying with stream of N_2_ gas to complete one cycle of LbL) for 5 cycles of LbL to prevent the infiltration of TCNQ within Cu-BPyDC thin film and afterwards, 15 consecutive LbL cycles with dipping time of 30 min were performed to grow Cu-TCNQ on top of Cu-BPyDC having well-defined interface (thickness values were estimated to be approximately 1 µm)^24^.

### Fabrication of TCNQ@Cu-BPyDC thin film

Prefabricated pristine Cu-BPyDC thin film was immersed into a saturated ethanolic solution of TCNQ for 48 h followed by washing with ethanol to remove the surface adsorbed TCNQ and dried under a stream of N_2_ gas^35^.

### Characterizations

The surface morphologies of both pristine and hetero-structured thin films were performed by Zeiss Ultra Plus FESEM. Contact angle measurements were measured using Holmarc’s Contact Angle Meter. Out-of-plane XRD data were recorded at room temperature using a Bruker D8 Advance diffractometer using Cu Kα radiation (λ = 1.5406 Å). Rietveld refinement of the PXRD patterns were carried out by a standardized procedure using the FullProf program^53,54^. Raman spectra (λ_exc_ = 632.8 nm) were recorded at Raman microscope (LabRAM HR, HoribaJobinYvon) with a 50× objective lens (spectral resolution of the system is ~1 cm^−1^). Solid state UV-vis absorption spectra were recorded on Shimadzu UV 3600 UV–Vis-NIR spectrophotometer. The XPS data were collected using Thermo Fisher Scientific ESCALAB Xi+. Electrical transport measurements (*I*–*V*) on various thin film samples were carried out using a Keithley 4200 SCS Parameter Analyzer system attached to an Everbeing probe station with a eutectic gallium indium (EGaIn) alloy as the contact electrode^21,24^ which is similar to earlier explored Hg-drop method for thin films of MOFs^22,55^. Temperature-dependent *I*–*V* measurements were recorded with the help of a conventional thermal chuck (from room-temperature to high-temperature) and a home-built liquid-N_2_ cooling set-up (from low-temperature to room-temperature) where standard Pt-100 sensor was used to monitor the temperature.

### Computational studies

The vibrational characteristics of molecular fragments in charge-neutral configurations were investigated using ab-initio DFT as implemented in Gaussian09 software. We have used the B3LYP hybrid functional with a 6–31 g basis set for each constituent atom and the Raman spectra were extracted from the frequencies obtained from the second derivative of the energy with respect to the nuclear position and a scaling factor of 30 cm^−1^ was consistently adopted^34^.

The geometrical optimizations, as well as the extraction of electronic structure information by considering Cu-BPyDC (Phase-I) as the bottom layer and Cu-TCNQ as the top layer were done by using Vienna Ab-initio Simulation Package (VASP) with projector augmented wave method^37^. The Perdew–Burke–Ernzerhof (PBE) functional of generalized gradient approximation was employed as exchange-correlation functions^37^. In order to describe the strongly correlated character of the Cu-3*d* orbitals accurately the DFT + U method was employed in the calculation with *U* value as 5 eV and *J* value as 1 eV (*U*_eff_ = 4.0 eV). All the above-mentioned structures are energy optimized using conjugat gradient method until the interatomic forces are reduced to 0.05 eV/ Å. The Brillouin zone integrations were performed using 3×3×5 and 4×4×2 Monkhorst–Pack K-point grids for bulk Cu-TCNQ (C_48_H_16_N_16_Cu_4_) and bulk Cu-BPyDC (C_48_H_56_N_8_O_32_Cu_4_) systems, respectively. Next, we carefully obtained the detailed electronic behavior of the interface in three-step process: (i) in the first step, interfacial distance between slabs are optimized and for this purpose, we relaxed the atomic positions of all the atoms in the top layer of Cu-TCNQ only (keeping positions of atoms in the bottom layer of Cu-BPyDC fixed) by using selective dynamics method; (ii) in the second step, we relaxed all the atoms in the unit cell to obtain the energy-optimized geometric structure of the interface; and (iii) finally, the electronic behavior of the Cu-TCNQ/Cu-BPyDC (C_236_H_200_N_48_O_64_Cu_16_) interface was calculated from the energy-optimized structure for which a 3×3×1 Monkhorst–Pack K-point grid was used. To estimate the proper electronic band structure, we further carried out hybrid functional calculations on top of normal PBE calculation using HSE06^38^ functionals. Here we have used 20 percent of Hartree–Fock exchange potential to parametrized the exchange correlation functional. Bader charge analysis:^39^ total number of valance electrons contributing to the Bader charge on Cu(I) structure (top layer) = 874; total number of valance electrons contributing to the Bader charge on Cu(II) structure (bottom layer) = 1048; and the total number of valance electrons contributing to the Bader charge on Cu(I)/Cu(II) interface = 1922.

Similarly, DFT(+U) calculations were carried out with the phase-II structure (minor component in our thin film) of Cu-BPyDC as the bottom layer and Cu-TCNQ as the top layer. Structures were energy optimized using conjugat gradient method until the interatomic forces are reduced to 0.01 eV/Å. The Brillouin zone integrations were performed using 3×3×5, Monkhorst–Pack grid for bulk Cu-TCNQ (C_48_H_16_N_16_Cu_4_) and bulk Cu-BPyDC (C_96_H_64_N_16_O_44_Cu_10_) system. Interface structure is made considering the configurations with minimum lattice mismatch and minimum strain on the individual surfaces, staking along (001) plane, for the hetero-structure Cu-TCNQ/Cu-BPyDC (C_480_H_224_N_128_O_88_Cu_32_). The top Cu-TCNQ layer of the interface was relaxed by keeping the bottom Cu-BPyDC undisturbed using selective dynamics to obtain an optimized interfacial distance. To estimate the proper density of states, we have used 3×3×1 Monkhorst–Pack grid and PBE functional. Furthermore, Bader charge analysis were carried out using Henkelman’s Group program and the analysis showed the total number of valance electrons contributing to the Bader charge on Cu(I) structure (top layer) = 1860; total number of valance electrons contributing to the Bader charge on Cu(II) structure (bottom layer) = 1804; and total number of valance electrons contributing to the Bader charge on Cu(I)/Cu(II) interface = 3664.

## Supplementary information

Supplementary Information

## Data Availability

All data supporting the findings of this study are available within this article and its Supplementary Information, as well as from the corresponding author upon request.
