# Publisher Correction: Charge-transfer interface of insulating metal-organic frameworks with metallic conduction

**DOI:** 10.1038/s41467-023-35887-5

**Published:** 2023-01-12

**Authors:** Pooja Sindhu, K. S. Ananthram, Anil Jain, Kartick Tarafder, Nirmalya Ballav

**Affiliations:** 1 Department of Chemistry, Indian Institute of Science Education and Research, Dr. Homi Bhabha Road, Pune, 411 008 India; 2 Department of Physics, National Institute of Technology Karnataka, Surathkal, Mangalore, 575 025 India; 3 Solid State Physics Division, Bhabha Atomic Research Centre, Mumbai, 400085 India; 4 Homi Bhabha National Institute, Anushakti Nagar, Mumbai, 400094 India

**Keywords:** Chemistry, Metal-organic frameworks

Correction to: *Nature Communications* 10.1038/s41467-022-35429-5, published online 12 December 2022

The original version of this Article incorrectly made the peer review reports for this paper available. In the correct HTML version, the link to the peer review file has been removed. The second sentence of the Peer Review Information section, which stated ‘Peer reviewer reports are available’ has been removed. This has been corrected in both the PDF and HTML versions of the Article.

