# Supplementary material for: Charge-transfer interface of insulating metal-organic frameworks with metallic conduction
Source: Nat Commun. 2022 Dec 12;13:7665. doi: 10.1038/s41467-022-35429-5 (PMC9744856; doi:10.1038/s41467-022-35429-5)
Supplement: Supplementary file 1 — Supplementary Information [file 41467_2022_35429_MOESM1_ESM.pdf]

Supplementary Information for

Charge-transfer interface of insulating metal-organic frameworks with metallic conduction

Pooja Sindhu[1], Ananthram K S[2], Anil Jain,[3,4] Kartick Tarafder[2], Nirmalya Ballav[1*]

[1]Department of Chemistry, Indian Institute of Science Education and Research, Dr. Homi Bhabha Road, Pune – 411 008, India

[2]Department of Physics, National Institute of Technology Karnataka, Surathkal, Mangalore – 575 025, India.

[3]Solid State Physics Division, Bhabha Atomic Research Centre, Mumbai – 400085, India

[4]Homi Bhabha National Institute, Anushakti Nagar, Mumbai – 400094, India

*Corresponding author. Email: nballav@iiserpune.ac.in

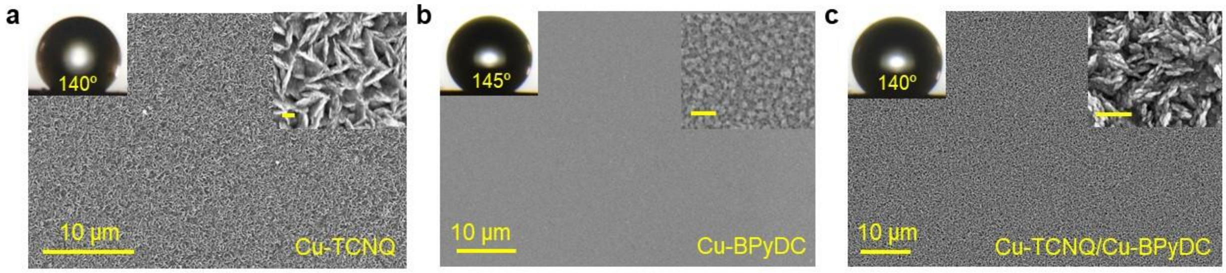

Supplementary Fig. 1. FESEM images of (a) pristine Cu-TCNQ, (b) pristine Cu-BPyDC and (c) hetero-structured Cu-TCNQ/Cu-BPyDC thin films showing the uniform coverage in each sample (inset: optical images of water contact angle (top left) and zoomed-in FESEM images at the scale of 200 nm (top right)).

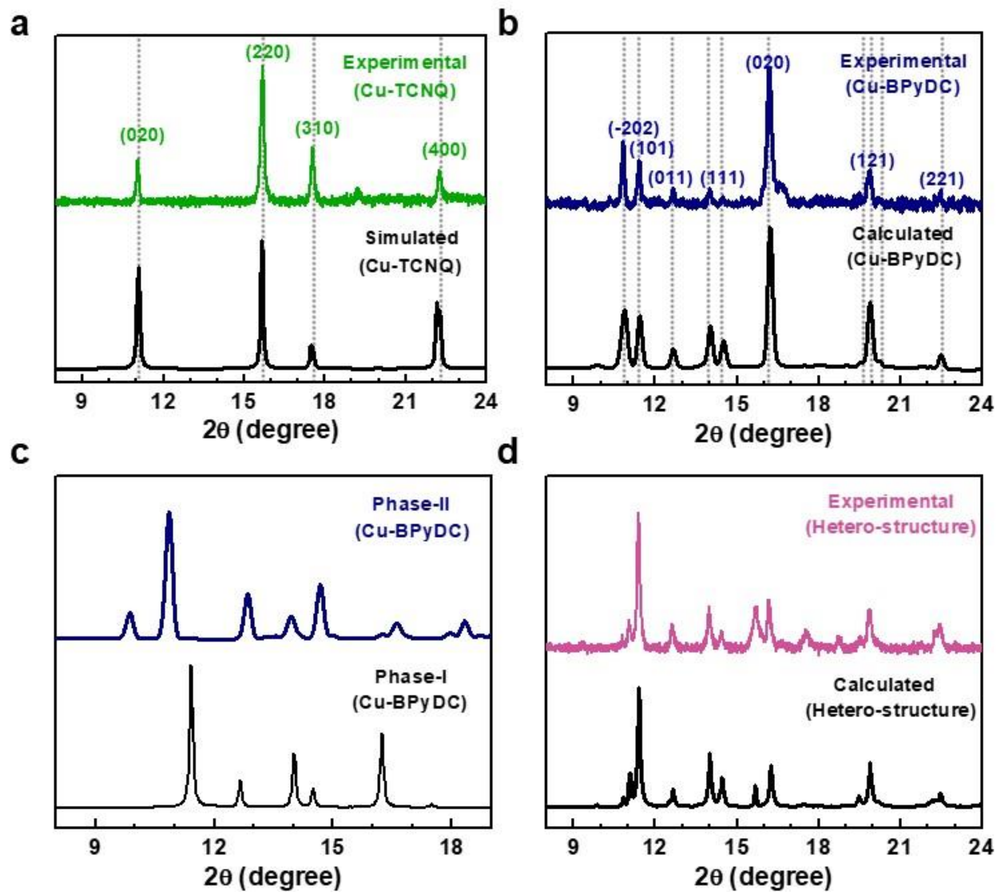

Supplementary Fig. 2. (a) Experimental XRD pattern of Cu-TCNQ thin film and simulated XRD pattern of Cu-TCNQ (crystal structure data was obtained from the supplementary files of Ref. 31). (b) Experimental XRD pattern of Cu-BPyDC thin film and calculated XRD pattern from the Rietveld refinement. For the Rietveld refinement of Cu-BPyDC, a mixed-phase was adopted, starting structural parameters for phase-I and Phase-II were used from Ref. 32, CCDC-710197 and Ref. 33, CCDC-1020991, respectively. The parameters were refined to match the observed and calculated XRD intensities. The refinement shows ratio of phase-I and phase-II as 84.23% and 15.77 %, respectively. Cell parameters for phase-I: a = 14.72(2) Å, b = 10.90(1) Å, c = 9.09(1) Å, $\alpha = \beta = \gamma = 90^{o}$, space group Pnna. Cell parameters for phase-II: a = 17.65(3) Å, b = 7.08(2) Å, c = 26.27(4) Å, $\alpha = 90^{o}$, $\beta = 106.4^{o}$, $\gamma = 90^{o}$, space group P1. (c) XRD patterns of phase-I and phase-II structures of Cu-BPyDC. (d) Experimental XRD pattern of the hetero-structured Cu-TCNQ/Cu-BPyDC thin film and the calculated XRD pattern from the Rietveld refinements.

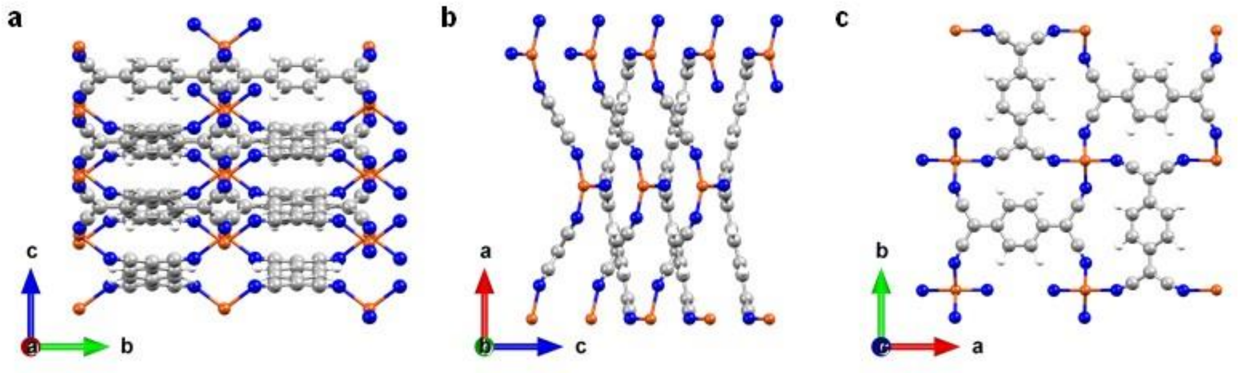

Supplementary Fig. 3. Insights into structure of Cu-TCNQ; view along (a) a-axis (b) b-axis and (c) c-axis; orange, grey, blue, and white colors represent copper, carbon, nitrogen, and hydrogen atoms, respectively.

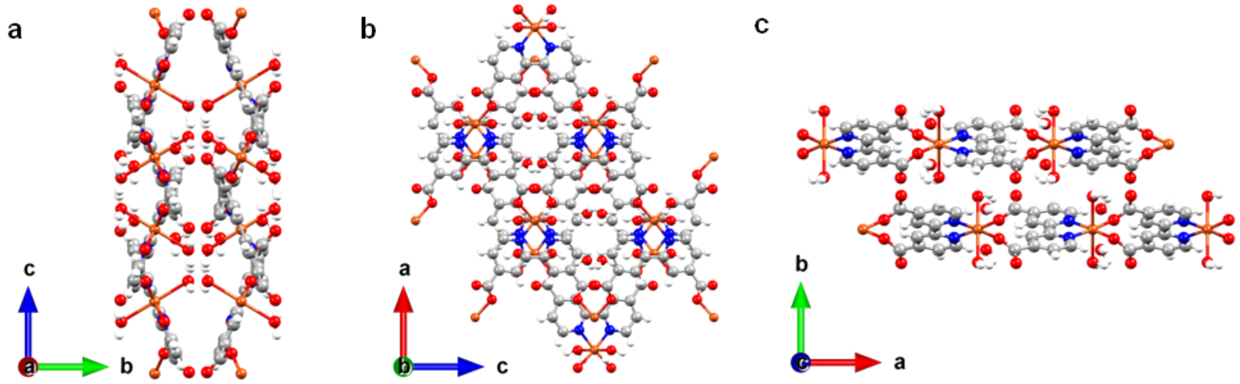

Supplementary Fig. 4. Insights into phase-I structure of Cu-BPyDC (Phase-I); view along (a) a-axis (b) b-axis and (c) c-axis; orange, grey, red, blue, and white colors represent copper, carbon, oxygen, nitrogen, and hydrogen atoms, respectively.

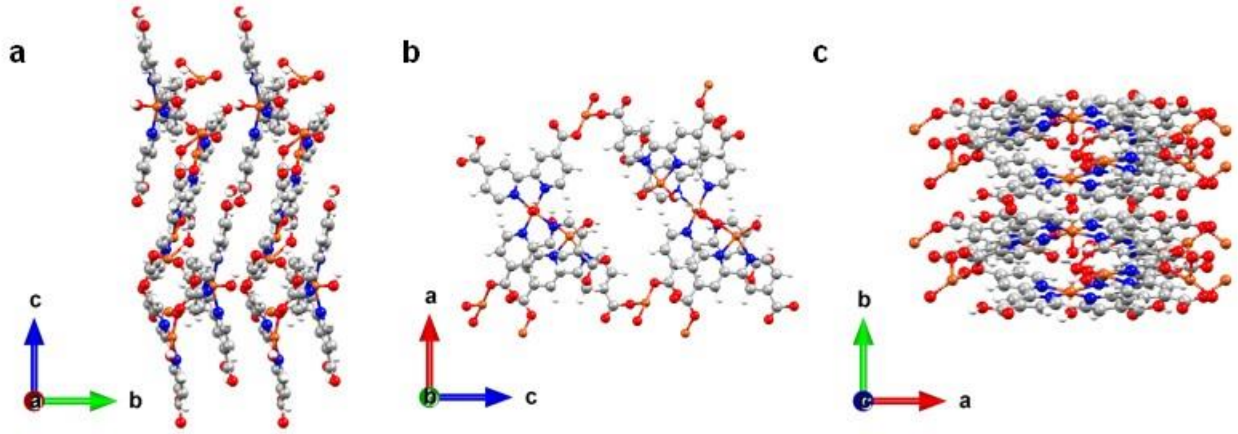

Supplementary Fig. 5. Insights into phase-II structure of Cu-BPyDC; view along (a) a-axis (b) b-axis and (c) c-axis; orange, grey, red, blue, and white colors represent copper, carbon, oxygen, nitrogen, and hydrogen atoms, respectively.

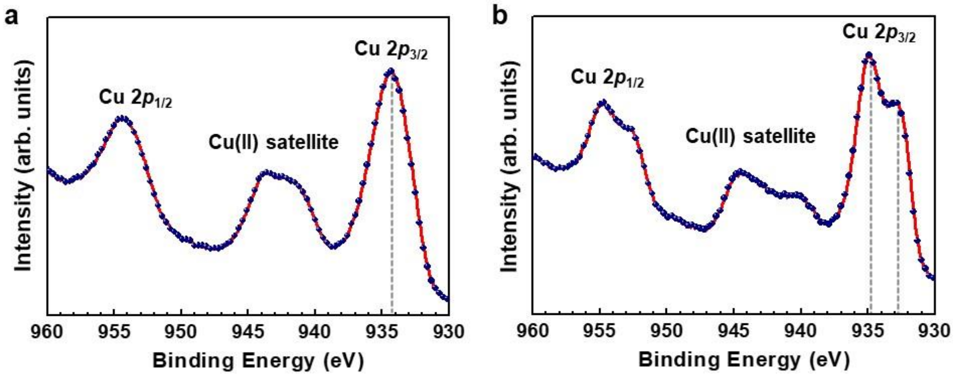

Supplementary Fig. 6. (a) Cu 2*p* XPS data recorded on pristine Cu-BPyDC thin film showing the Cu 2$p_{3/2}$ signal at ~934.2 eV along with strong satellite feature clearly evidencing the presence of Cu(II) in the thin film. (b) Cu 2*p* XPS data recorded on hetero-structured Cu-TCNQ/Cu-BPyDC sample where few layers of Cu-TCNQ (5 cycles) was grown on the pristine Cu-BPyDC thin film showing Cu 2$p_{3/2}$ signal at ~934.2 eV (Cu(II) species) and ~932.5 eV (Cu(I) species) – as indicated by dotted grey lines. As for the Cu 2*p* XPS data on pristine Cu-TCNQ thin film, please refer Ref. 27.

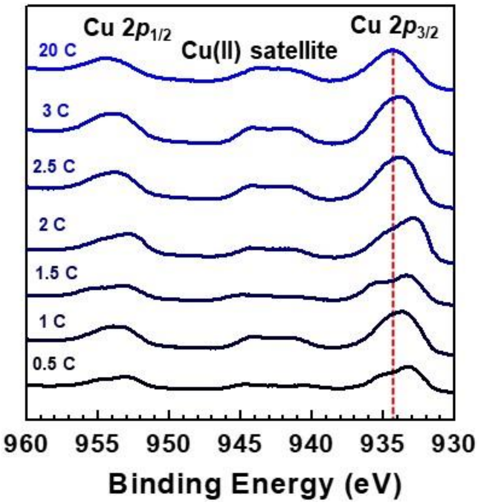

Supplementary Fig. 7. Cu 2*p* XPS spectra recorded during the growth of pristine Cu-BPyDC thin film at each step of LbL method from 0.5 to 3 cycles and at 20th cycle (C stands for cycle) with strong satellite feature clearly evidencing the presence of Cu(II) in the thin film. During the initial growth (up to 2 cycles), the Cu 2$p_{3/2}$ signals at binding energy value of ~933.1 eV and ~935.2 eV could be assigned to two distinctive coordination of Cu(II) ions to four N and four O atoms from BPyDC ligands, respectively, in phase-II structure of Cu-BPyDC. After 2 cycles (up to 3 cycles), the Cu 2$p_{3/2}$ signal at binding energy value of ~933.8 eV could be assigned to mixed phase-I and phase-II structures of Cu-BPyDC (note that in phase-I structure of Cu-BPyDC, Cu(II) ion is coordinated simultaneously to two N and two O atoms from BPyDC ligands). After 20 cycles, the Cu 2$p_{3/2}$ signal at binding energy value of ~934.2 eV could originate predominantly from the phase-I structure of Cu-BPyDC. Therefore, cycling dependent XPS data complemented the cycling dependent Raman spectra as well as XRD patterns.

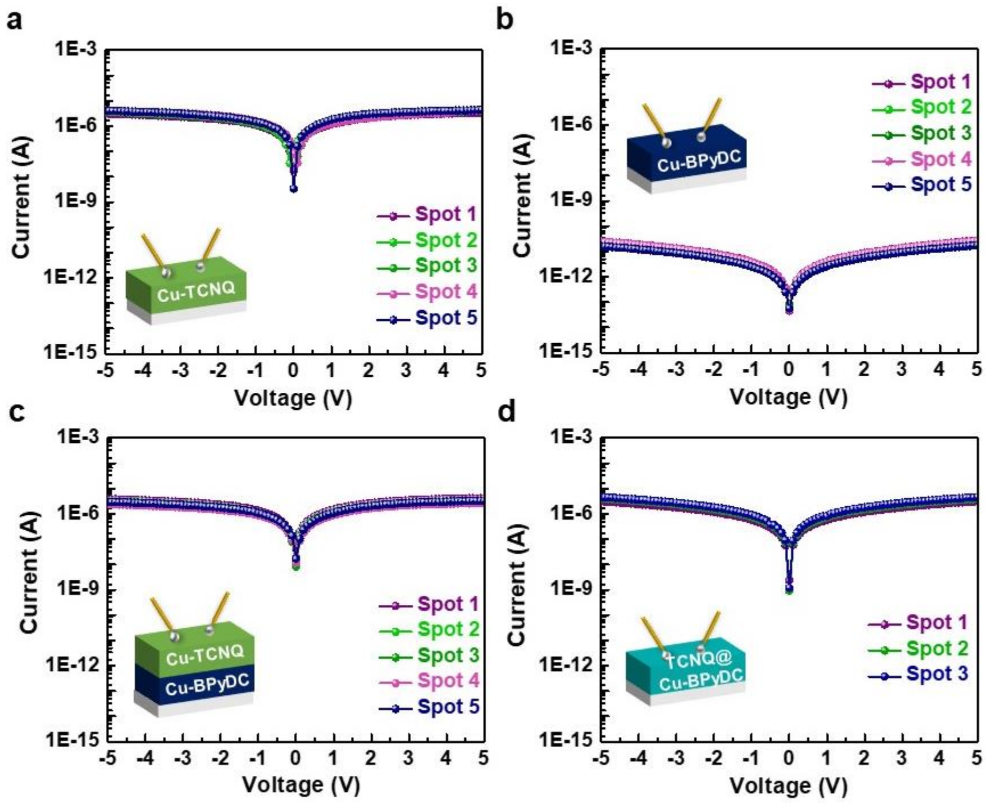

Supplementary Fig. 8. In-plane current-voltage (*I*-*V*) characteristics of (a) pristine Cu-TCNQ (b) pristine Cu-BPyDC, (c) hetero-structured Cu-TCNQ/Cu-BPyDC, and (d) TCNQ@Cu-BPyDC thin films on various spots in each sample. Insets: schematic of measurement with eutectic GaIn contacts.

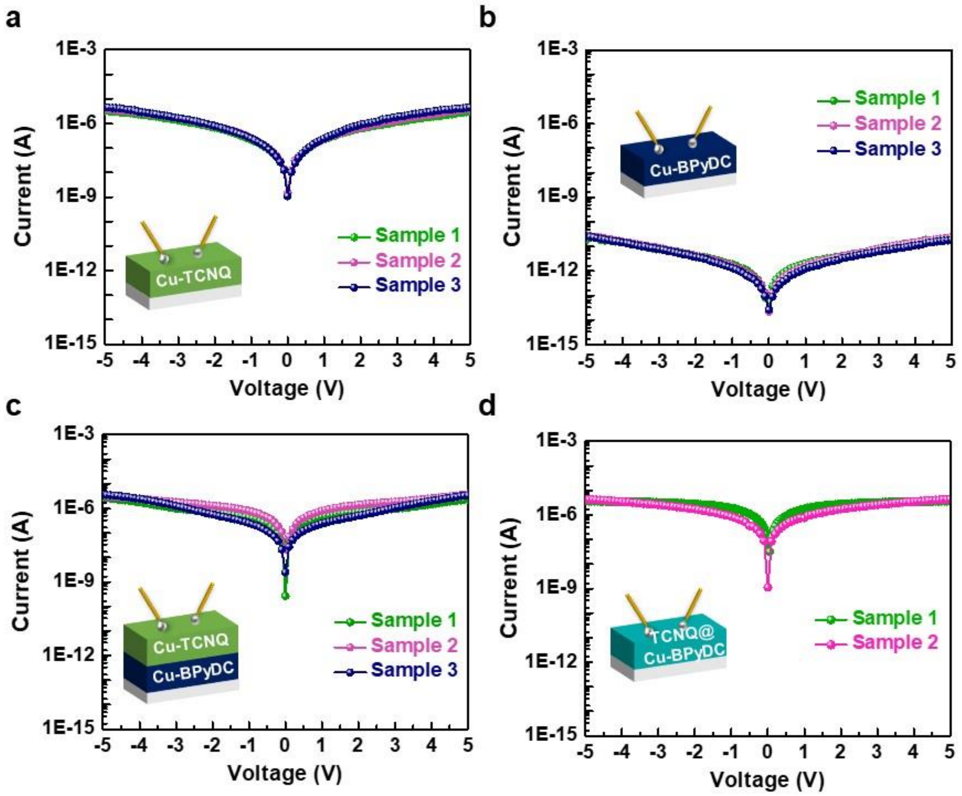

Supplementary Fig. 9. In-plane current-voltage (*I*-*V*) characteristics of (a) pristine Cu-TCNQ (b) pristine Cu-BPyDC (c) heterostructured Cu-TCNQ/Cu-BPyDC and (d) TCNQ@Cu-BPyDC thin films across different batches of samples. Insets: schematic of measurement with eutectic GaIn contacts.

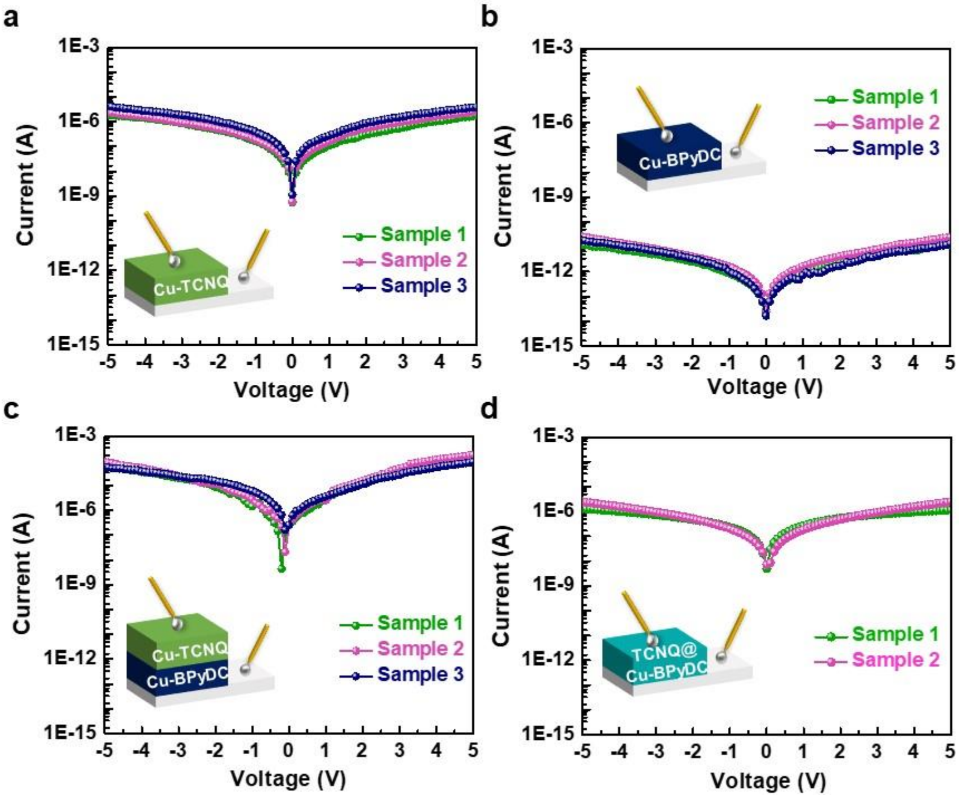

Supplementary Fig. 10. Cross-plane current-voltage (*I*-*V*) characteristics of (a) pristine Cu-TCNQ (b) pristine Cu-BPyDC (c) heterostructured Cu-TCNQ/Cu-BPyDC and (d) TCNQ@Cu-BPyDC thin films across different batches of samples. Insets: schematic of measurement with eutectic GaIn contacts.

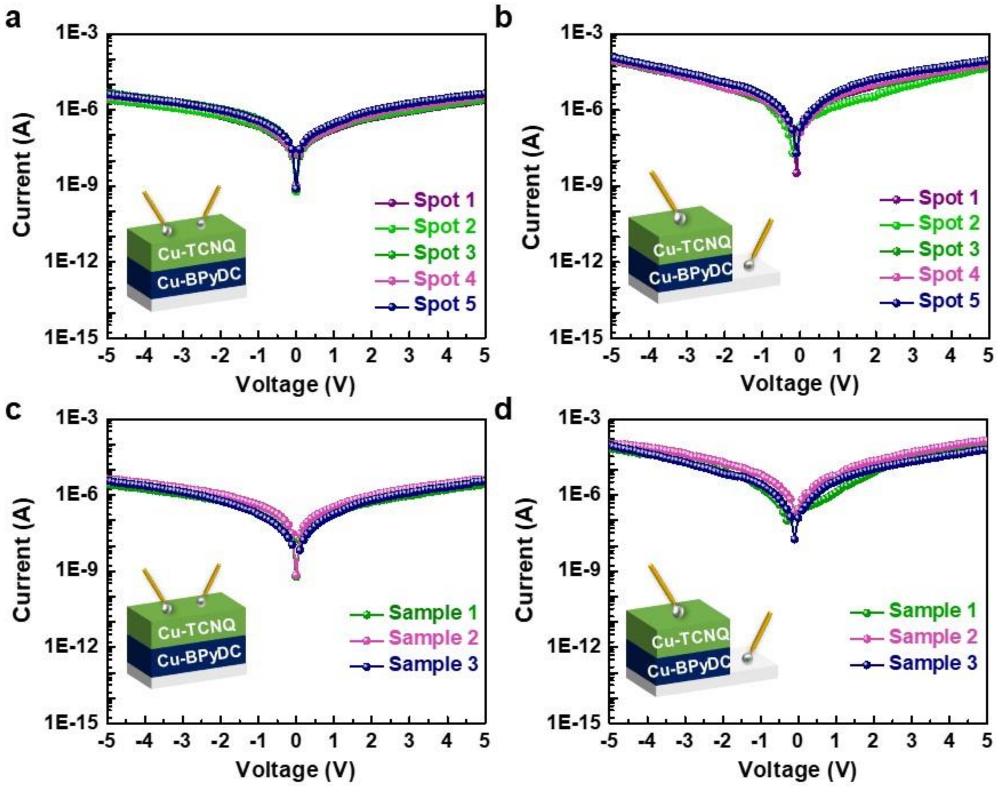

Supplementary Fig. 11. Current-voltage (*I*-*V*) characteristics recorded on 3 months old (kept at ambient conditions) hetero-structured Cu-TCNQ/Cu-BPyDC thin films - in (a) in-plane and (b) cross-plane modes on various spots in the sample and (c) in-plane and (d) cross-plane modes across different samples. Insets: schematic of measurement with eutectic GaIn contacts.

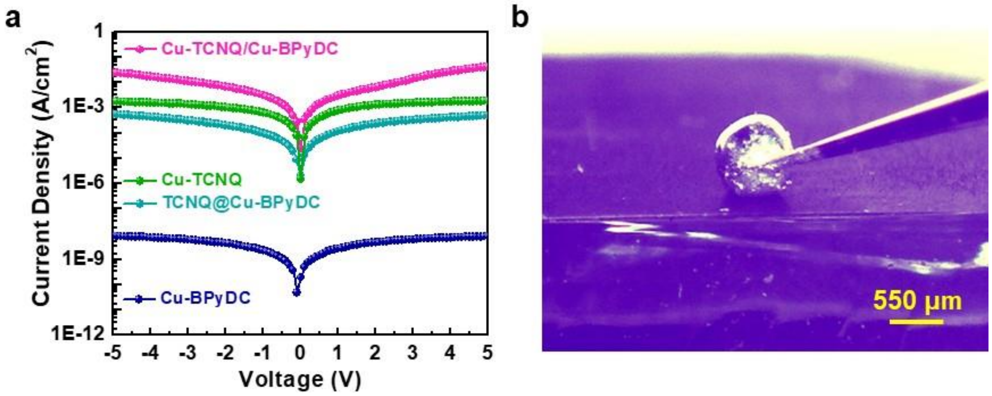

Supplementary Fig. 12. Cross-plane current density-voltage (*J*-*V*) characteristics (a) recorded on pristine Cu-TCNQ, pristine Cu-BPyDC, hetero-structured Cu-TCNQ/Cu-BPyDC, and TCNQ@Cu-BPyDC thin films; and photograph of EGaIn top contact electrode (b) showing the contact diameter of ca. 550 μm which was used to estimate the resistivity value (similar to the method outlined in Ref. 55). To directly compare the resistivity value of our samples with those in Ref. 22, we have taken the bias voltage at 0.5 V.

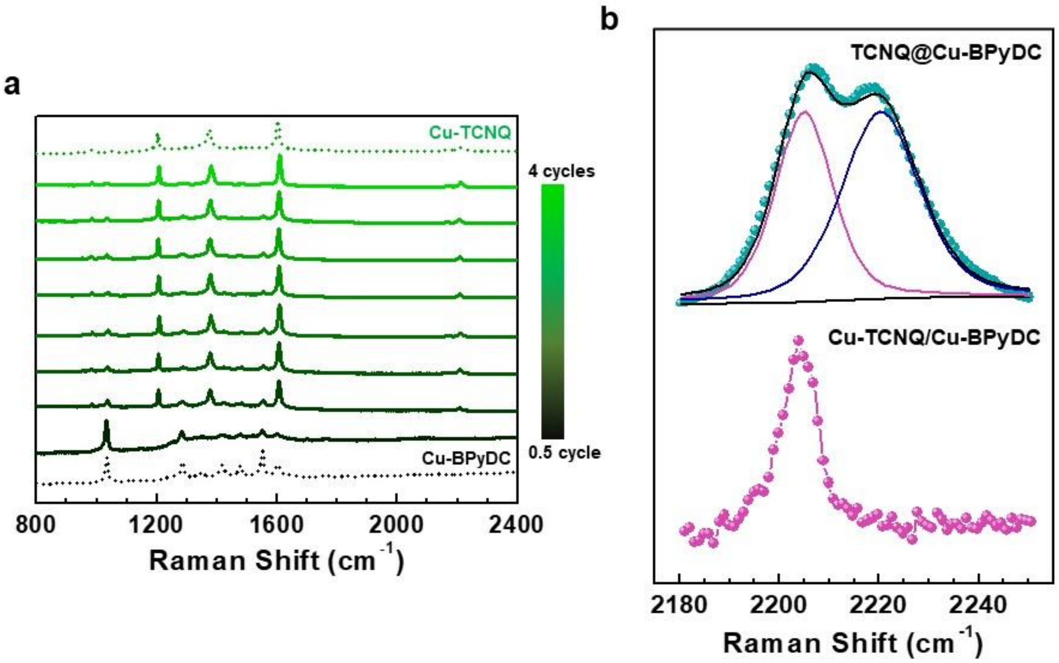

Supplementary Fig. 13. (a) Raman spectra recorded during the growth of Cu-TCNQ on top of Cu-BPyDC thin film, at each step of LbL and up to 4 cycles; and compared with the Raman spectrum of the pristine Cu-TCNQ thin film. (b) Fitted Raman spectrum of the TCNQ@Cu-BPyDC thin film and as comparison Raman spectrum of hetero-structured Cu-TCNQ/Cu-BPyDC thin film is provided.

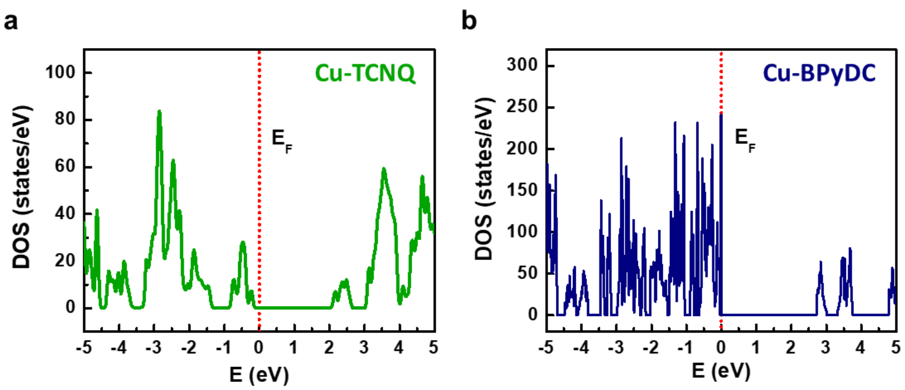

Supplementary Fig. 14. Total density of states (DOS) plots for (a) Cu-TCNQ (green solid line) and (b) Cu-BPyDC (blue solid line). Fermi energy (E_F) is marked by red dotted lines.

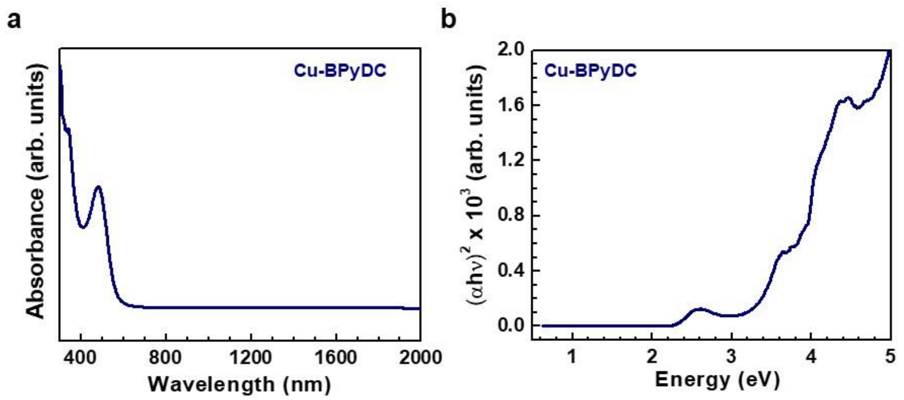

Supplementary Fig. 15. Solid-state UV-vis spectra of (a) pristine Cu-BPyDC thin film and (b) corresponding Tauc plot. As for the Tauc plot of pristine Cu-TCNQ, please refer Ref. 24.

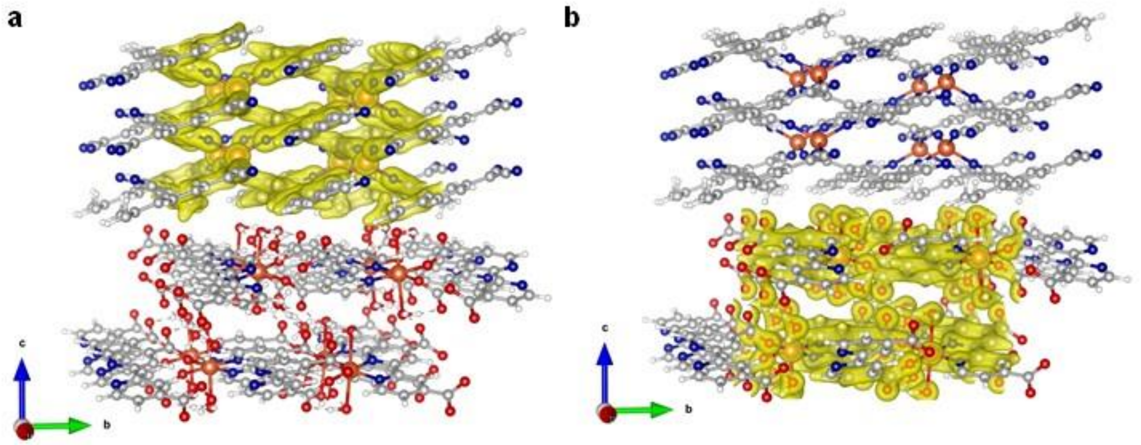

Supplementary Fig. 16. Total charge density plot for (a) only Cu-TCNQ in hetero-structured Cu-TCNQ/Cu-BPyDC system and (b) only Cu-BPyDC structure in hetero-structured Cu-TCNQ/Cu-BPyDC system. The atoms carbon, hydrogen, nitrogen, oxygen, and copper are represented by colors grey, white, blue, red, and orange, respectively.

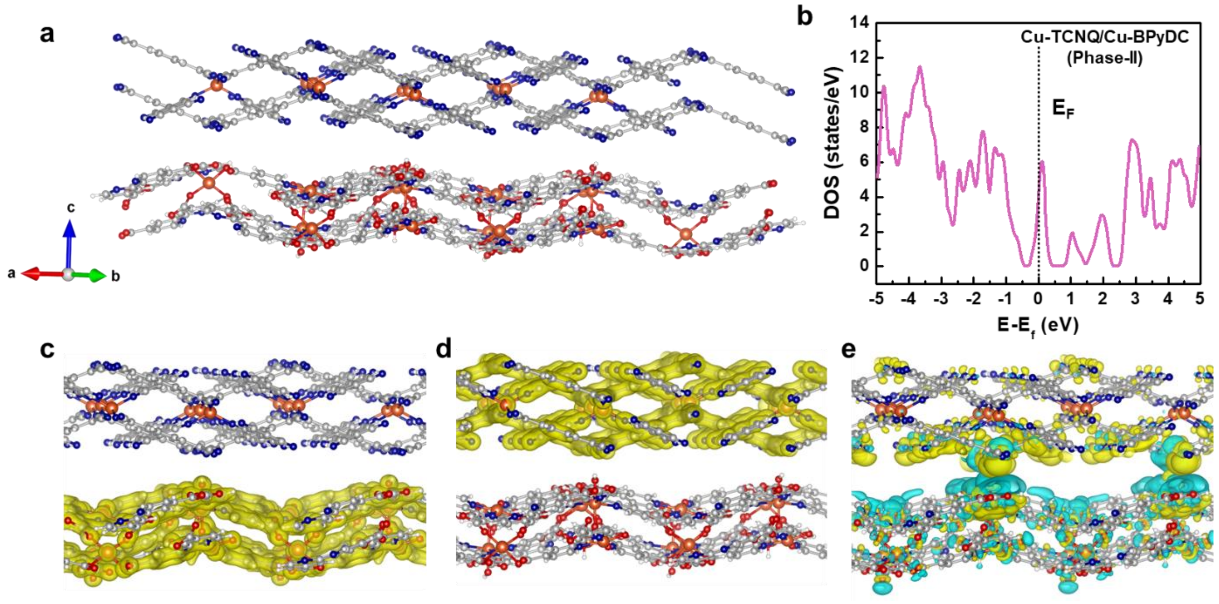

Supplementary Fig. 17. (a) Optimized interface geometric structure of Cu-TCNQ (top, $C_{48}H_{16}N_{16}Cu_4$) and phase-II Cu-BPyDC (bottom, $C_{96}H_{64}N_{16}O_{44}Cu_{10}$) interface where orange, grey, red, blue, and white colours represent copper, carbon, oxygen, nitrogen, and hydrogen atoms, respectively. (b) The total density of state (DOS) for the interface structure, a continuous state at the Fermi energy (E_F) appeared due to significant charge redistribution and strong hybridization at the interface. (c) Total charge density plot for only Cu-BPyDc (Phase-II) in hetero-structured Cu-TCNQ ($C_{48}H_{16}N_{16}Cu_4$)/Cu-BPyDC ($C_{96}H_{64}N_{16}O_{44}Cu_{10}$) system. (d) Total charge density plot for only Cu-TCNQ in hetero-structured Cu-TCNQ ($C_{48}H_{16}N_{16}Cu_4$)/Cu-BPyDC ($C_{96}H_{64}N_{16}O_{44}Cu_{10}$) system. (e) Percolated charge density due to the formation of the interface Cu-TCNQ/Cu-BPyDC ($C_{480}H_{224}N_{128}O_{88}Cu_{32}$) is presented where electron accumulation and electron depletion are represented by yellow and cyan isosurfaces, respectively.

Supplementary Table 1. Lattice constants information of all optimized structures.

Structure	a (Å)	b (Å)	c (Å)	α	β	γ	Space group/HM notation
**Cu-TCNQ** **$C_{48}H_{16}N_{16}Cu_4$**	15.53	16.47	3.806	90º	90º	90º	1/P1
**Cu-BPyDC (Phase-I)** **$C_{48}H_{56}N_8O_{32}Cu_4$**	17.35	14.89	30.60	90º	90º	90º	52/Pnna
**Cu-TCNQ/Cu-BPyDC (Phase-I) interface** **$C_{236}H_{200}N_{48}O_{64}Cu_{16}$**	15.60	15.17	30.48	90º	90º	90º	-nil-

Supplementary Table 2. Atoms-wise total Bader charge contribution.

Atom	Charge (electron) in the Layer					
	Total charge on atoms in the top-layer per unit cell [8 Cu atoms and 32 N atoms are considered]		Net charge difference per atom due to formation of interface	Total charge on atoms in the bottom-layer per unit cell [8 Cu atoms, 32 N atoms and 64 O atoms are considered]		Net charge difference per atom due to formation of interface
	Interface	Individual		Interface	Individual	
**Cu**	82.71	80.74	0.24	78.78	79.27	-0.06
**O**	- nil -	- nil -	- nil-	444.30	461.78	-0.27
**N**	177.33	199.12	-0.68	95.36	99.71	-0.27

Supplementary Table 3. Details of charge (electron) transferred from different atoms at the interface.

**Atoms [B & T indicate bottom and top layers in the unit cell]**	**Total charge on the atoms in the Cu(I)/Cu(II) interface**	**Charge on the atom in the individual layer**	**Net Bader charge at the interface**
**Cu-T (4 atoms)**	40.36	41.35	-0.99
**Cu-B (4 atoms)**	39.64	39.44	0.20
**N-T (8 atoms)**	49.82	47.25	2.57
**N-B (8 atoms)**	49.8	47.62	2.18
**O-B (4 atoms)**	28.09	28.53	-0.44
**Total effective charge at the interface**			**3.52**

Supplementary Table 4. Lattice constants information of all optimized structures.

**Structure**	**a (Å)**	**b (Å)**	**c (Å)**	**α**	**β**	**γ**	**Space group/HM notation**
**Cu-TCNQ $C_{48}H_{16}N_{16}Cu_4$**	15.53	16.47	3.806	90°	90°	90°	1/P1
**Cu-BPyDC (Phase-II) $C_{96}H_{64}N_{16}O_{44}Cu_{10}$**	16.72	6.419	24.03	90°	104°	90°	1/P1
**Cu-TCNQ/Cu-BPyDC (Phase-II) interface $C_{480}H_{224}N_{128}O_{88}Cu_{32}$**	34.63	23.43	25.50	90°	90°	104.5°	1/P1

Supplementary Table 5. Atoms-wise total Bader charge contribution by considering Cu-BPyDC (Phase-II) structure.

**Atom**	**Charge (electron) in the Layer**					
	**Total charge on atoms in the top-layer per unit cell [8 Cu atoms and 32 N atoms are considered]**		**Net charge difference per atom due to formation of interface**	**Total charge on atoms in the bottom-layer per unit cell [8 Cu atoms, 32 N atoms and 64 O atoms are considered]**		**Net charge difference per atom due to formation of interface**
	**Interface**	**Individual**		**Interface**	**Individual**	
**Cu**	120.6684	120.6092	0.0592	201.3270	201.3092	0.0178
**O**	- nil -	- nil -	- nil-	603.2546	603.4929	-0.2383
**N**	562.9860	563.2321	-0.2461	184.08198	184.3694	-0.2874

Supplementary Table 6. Details of charge (electron) transferred from different atoms at the interface from Cu-TCNQ and Cu-BPyDC (Phase-II) as top and bottom layers respectively.

**Atoms [B & T indicate bottom and top layers in the unit cell]**	**Total charge on the atoms in the Cu(I)/Cu(II) interface**	**Charge on the atom in the individual layer**	**Net Bader charge at the interface**
**Cu-T (12 atoms)**	120.66845	120.60922	0.06
**Cu-B (10 atoms)**	100.545645	100.216273	0.33
**N-T (12 atoms)**	69.767518	69.710702	0.06
**N-B (12 atoms)**	69.227223	69.315718	0.09
**O-B (18 atoms)**	123.720431	123.797303	0.08
**Total effective charge at the interface**			**0.62**
